# Identification of the NAC Transcription Factors and Their Function in ABA and Salinity Response in *Nelumbo nucifera*

**DOI:** 10.3390/ijms232012394

**Published:** 2022-10-16

**Authors:** Shuping Zhao, Tao Jiang, Yao Zhang, Kailing Zhang, Kai Feng, Peng Wu, Liangjun Li

**Affiliations:** 1 School of Horticulture and Plant Protection, Yangzhou University, Yangzhou 225009, China; 2 Joint International Research Laboratory of Agriculture and Agri-Product Safety of Ministry of Education of China, Yangzhou University, Yangzhou 225009, China

**Keywords:** NAC transcription factor, salinity, ABA, gene expression, *Nelumbo nucifera* Gaertn

## Abstract

*Nelumbo nucifera* Gaertn. is an important perennial aquatic herb that has high ornamental, edible, medicinal, and economic value, being widely distributed and used in China. The NAC superfamily (NAM, ATAF1/2, CUC2) plays critical roles in plant growth, development, and response to abiotic and biotic stresses. Though there have been a few reports about NAC genes in lotus, systematic analysis is still relatively lacking. The present study aimed to characterize all the *NAC* genes in the lotus and obtain better insights on the *NnNACs* in response to salt stress by depending on ABA signaling. Here, 97 *NAC* genes were identified by searching the whole lotus genome based on the raw HMM models of the conserved NAM domain and NAC domain. They were characterized by bioinformatics analysis and divided into 18 subgroups based on the phylogenetic tree. Cis-element analysis demonstrated that *NAC* genes are responsive to biotic and abiotic stresses, light, low temperature, and plant hormones. Meanwhile, *NAC* genes had tissue expression specificity. qRT-PCR analysis indicated that *NAC* genes could be upregulated or downregulated by NaCl treatment, ABA, and fluoridone. In addition, *NAC016*, *NAC025*, and *NAC070*, whose encoding genes were significantly induced by NaCl and ABA, were located in the nucleus. Further analysis showed the three NAC proteins had transcriptional activation capabilities. The co-expression network analysis reflected that NAC proteins may form complexes with other proteins to play a role together. Our study provides a theoretical basis for further research to be conducted on the regulatory mechanisms of salinity resistance in the lotus.

## 1. Introduction

Plant transcription factors (TFs) play crucial roles in plant growth, development, and abiotic and biotic responses [1]. Considering the importance of TFs in responding to various stimuli, analyzing their function mechanism is becoming indispensable for vegetable crop breeding. With more plant genomes sequenced and transgenic technology developed, a large number of TFs have been identified, and their functions have been determined. NAC (non-apical meristem (NAM), Arabidopsis transcription activation factor (ATAF), and calathiform cotyledon (CUC)) transcription factors characterized by the presence of a highly conserved N-terminal NAC domain and diverse C-terminal domains have been widely studied [2,3]. The N-terminal NAC domain consists of about 160 amino acids that could bind to the *cis*-elements of their target genes, and C-terminal domains are a fundamental area to interact with other proteins and regulate other gene expression [4,5]. The structure of NAC proteins being well-identified provided a framework for further understanding the functions of NAC proteins at the transcriptional level. Subsequently, research on the functions of NAC transcription factors mushroomed in a variety of species.

Gene family analysis as a basic method plays an important role in investigating the functions of plant transcription factors. The members of the NAC family have been identified and reported in many species by gene family analysis. For instance, 108 members have been identified in *Arabidopsis*, 151 members in rice, 150 members in soybean, 261 members in *M. sinensis*, 132 members in peanut, 151 members in sunflower, 87 members in maize, 111 members in celery, 104 members in pepper, and 110 members in potato [6,7,8,9,10,11,12]. NAC proteins have not only been identified, but their functions have also been well studied. NAC TFs play important roles in many vital biological processes and plant stress responses [13,14,15,16,17,18]. 

Considering that abiotic stresses could adversely affect crop growth and productivity, it is very important to study the functions of NAC TFs in improving crop resistance to abiotic stresses, which have been well elucidated in many species in last decade. In grain crop rice, *ONAC066*, *ONAC095*, *OsNAC2*, *OsNAC006*, *OsNAC67*, *OsNAC016*, and *OsNAP* function as positive or negative regulators of the drought stress response [15,19,20,21,22,23,24]. In vegetable crops, NAC TF genes, such as *JUB1*, *SlVOZ1*, *SlNAC35*, *StNAC053*, and *CaNAC46*, also play an important role in plant responses to drought stresses [25,26,27,28,29]. In addition, NAC TFs have been widely focused on regarding plant resistance to salinity, which reflects the fact that one NAC TF can have multiple functions. Potato *StNAC053*, grapevine *VvNAC17*, pepper *CaNAC46*, rice *ONAC022*, and wheat *TaNAC29* enhance not only plant drought tolerance but also salt tolerance [25,26,30,31,32]. These research works provide us with a clear research direction and a very important basis from which to elucidate the regulation mechanism of NAC TFs.

NAC TFs can regulate several plant signal networks and be regulated by environmental stimuli, plant hormones, and miRNAs. Environmental stimuli, such as light, temperature, drought, salinity, water, etc., could induce the expression of NAC TFs [33,34,35]. Furthermore, plant hormones are the essential regulators of plant growth and development. The interplay between plant hormones and NAC TFs has become an interesting research topic. Wu et al. discussed interaction between NAC TFs and ethylene and abscisic acid (ABA) pathways in controlling tomato fruit development and ripening [36]. *OsNAC3, OsNAC45*, and *VvNAC17* improved transgenic plant ABA sensitivity and salt tolerance [31,37,38]. Further study demonstrated that CrNAC036 can directly bind and negatively regulate *CrNCED5* expression to hinder ABA biosynthesis in citrus [39]. Interestingly, recent studies showed microRNAs (miRNAs) comprising about 22 nt endogenous noncoding RNAs could take part in plant abiotic responses by targeting *NAC* genes. In other words, *NAC* genes were regulated by plant miRNAs. Peu-miR164 regulated its target genes *PeNAC070* and *PeNAC081*, which are involved in the poplar response to abiotic stress [40]. osa-miR164c negatively regulated *OsCUC1* and *OsCUC3*, which are involved in rice meristem/organ boundary specification [41]. In addition, miR1514a modulates *NAC700* transcription to trigger phasiRNA formation in response to water deficit in legumes [42]. These reports indicate that the NAC TF-related networks are complex and important for plant growth and development. 

Lotus (*Nelumbo nucifera* Gaertn.), popular ornamental, edible, and medicinal plant which belongs to the small family *Nelumbonaceae*, will be an important model plant in horticulture [43]. With three lotus genomes sequenced, the lotus TFs, such as AP2/ERF, WRKY, bHLH, and the MADS gene family, have been widely studied [44,45,46,47,48,49,50]. However, the NAC gene family has not yet been comprehensively studied in lotus. Though 82 *NnNAC* genes have been found using the PlantTFDB 4.0, the NAC gene family still needs to be identified by searching the whole lotus genome. Here, all NAC members of lotus were identified, and their distribution, phylogenetic relationship, gene structure, and synteny were analyzed. In addition, certain candidate NAC TFs which had a higher expression in lotus leaves were further studied though subcellular localization and expression level analysis by qRT-PCR. These results will further deepen the understanding of NAC TFs and provide several candidate genes related to salinity and abscisic acid (ABA) response in lotus.

## 2. Results

### 2.1. Identification of NAC Transcription Factors in Nelumbo nucifera

**Figure 1:**
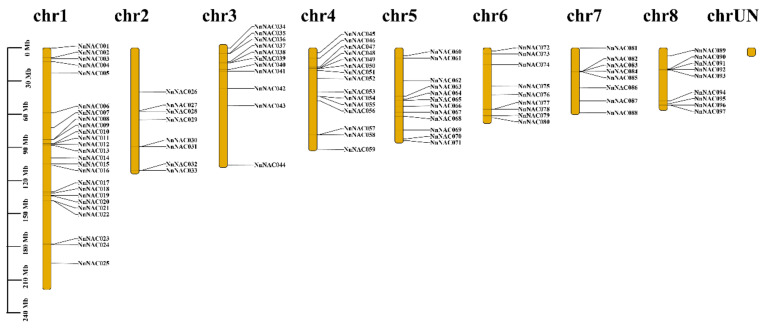
Chromosomal locations of lotus *NAC* genes. The brown sticks represent the chromosomes, and the chromosome numbers are shown on top of the sticks. The lengths of the chromosomes are on the left of the figure.

To identify all NAC TFs in *Nelumbo nucifera*, the conserved NAM domain (PF02365) and NAC domain (PF01849) were used to seek the respective raw HMM models. Based on these two HMM models, 107 and 106 candidate members were identified, and the redundant forms of the same genes were removed. In total, 97 NAC TF members were identified and named NAC001–NAC097 according to their chromosome location (Table 1 and Figure 1). The basic information, including genomic length, amino acid residues, *p*I, and molecular weight, was calculated. For example, the polypeptide lengths of the predicted NACs varied widely, ranging from 76 to 684. In addition, the p*I* data showed that NACs have both acidic and alkaline members. The information also contained the members’ gene IDs, which could help researchers obtain recent news on NACs (Table 1).

### 2.2. Phylogenetic Analysis and Classification of NAC Genes

**Figure 2:**
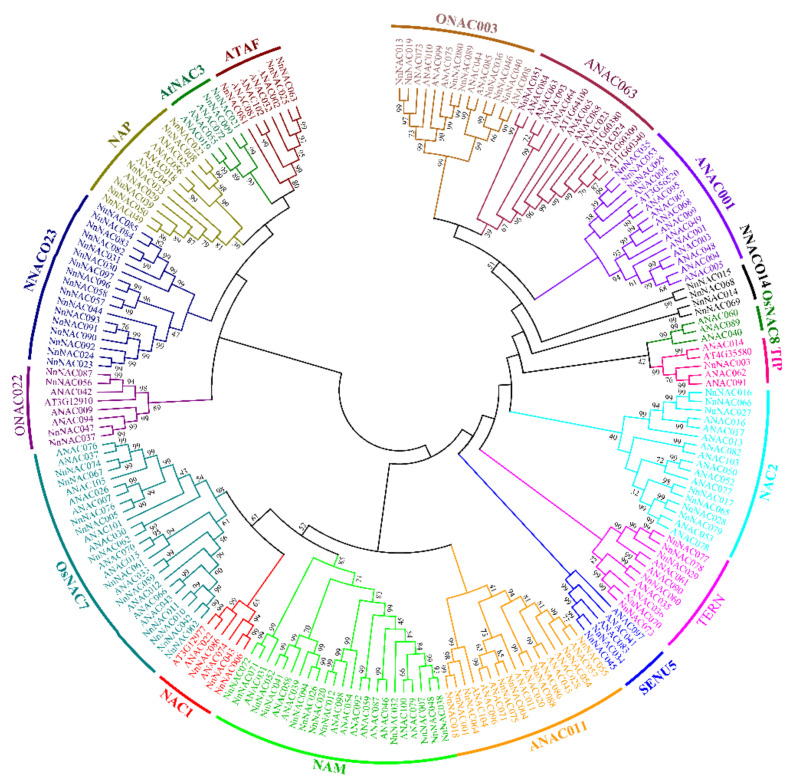
Neighbor-joining phylogenetic tree of the NAC family in lotus and *Arabidopsis*.

Eighteen NAC subgroups have been classified in rice and *Arabidopsis* [51]. In our results, 18 new subgroups were redefined in which no lotus NAC members belong to ONAC001 and OsNAC3. However, the lotus NAC members family had two specific subgroups named NNACO23 and NNAC014, which indicated lotus is species-specific compared to *Arabidopsis* and rice (Figure 2). 

### 2.3. Gene Structure and Motif Composition of the NAC Gene Family

**Figure 3:**
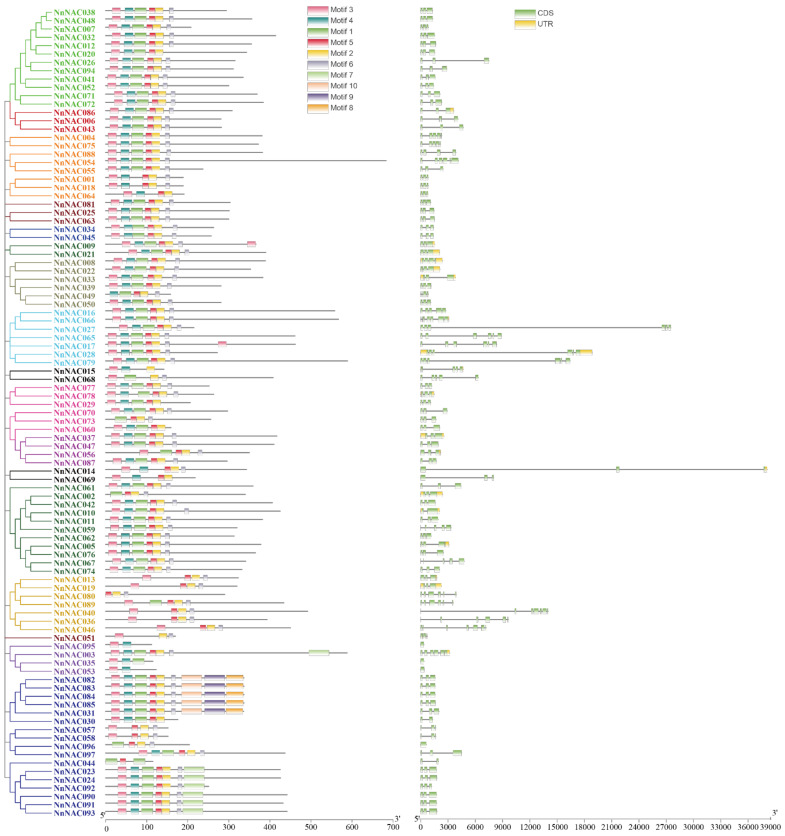
The structures of *NAC* genes and NAC proteins. The left represents the phylogenetic relationship of NAC genes. The middle represents the conserved motifs in NAC proteins, in which different colors represent different motifs. The right represents the intron–exon structures of *NAC* genes. The exons, introns, and untranslated regions (UTRs), are indicated by the green boxes, black lines, and yellow boxes, respectively.

The sequence features including introns, exons, and motifs were visualized by the TBtools software [52]. According to the frequency of occurrence, motifs 1–5 were the five most frequently presented motifs, covering almost all NAC members. Furthermore, motifs 8–10 only existed in the members NnNAC082, 083, 084, 085, and 031, which belong to subgroup NNAC023. Interestingly, NnNAC009 and NnNAC017 possessed two copies of motif 3. In addition, the motifs in subgroup ONAC003 showed a character of random arrangement. The exhibition of exon–intron structure showed that there is evolutionary diversity. Most of the members had more than three exons, and only four members had one exon. *NnNAC069*, *27*, *28*, and *79* possessed relatively longer intron lengths (Figure 3).

### 2.4. Cis-Acting Elements and Synteny Analysis of NAC Gene Promotors

**Figure 4:**
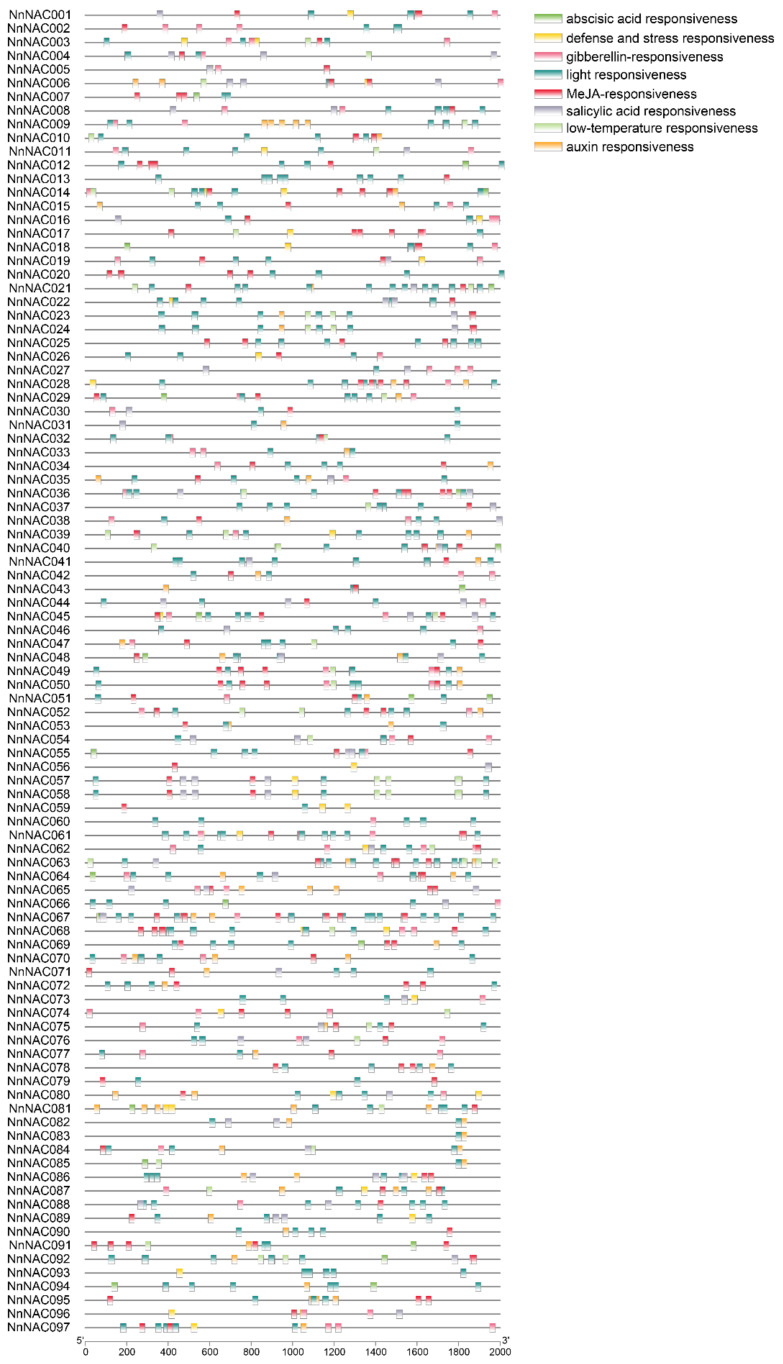
The cis-acting elements in the promoters of soybean SRS genes.

To investigate the functions of *NAC* genes, the cis-acting elements of *NAC* gene promotors were analyzed by the online tool PlantCARE; the data were then visualized by TBtools. All *NAC* genes had the “light responsiveness” element, suggesting that the “light responsiveness” element is fundamental for plant growth and development. In addition, plant hormones were the important factors for plant growth, development, and response to the environment. Furthermore, the plant hormone elements “abscisic acid responsiveness”, “gibberellin responsiveness”, “MeJA responsiveness”, “salicylic acid responsiveness”, and “auxin responsiveness” were widely presented in *NAC* gene promotors (Figure 4). The “defense and stress responsiveness” element, which weas also widely presented in *NAC* gene promotors, played important roles in plant responses to abiotic and biotic stresses. The “low-temperature responsiveness” element was also important because lotus root is occasionally affected by low temperatures. The above elements suggest that *NAC* genes in lotus root may function in diverse aspects of lotus root growth and development. To compare the evolutionary events between lotus and Arabidopsis, synteny analysis was carried out using the orthologous NAC gene pairs. A total of 69 collinear genes were retrieved from the two species (Figure S1).

### 2.5. Expression Patterns of NAC Genes in Different Plant Tissues

To investigate the tissue-specific expression of *NAC* genes, we obtained data from the “Nelumbo Genome Database” which contained the expression levels of *NAC* genes in the petiole, leaf, root, and cotyledon (Table S2). The results showed that *NAC* genes had tissue expression specificity, indicating that their functions were diverse. For example, *NAC006* was only expressed in the leaf, root, and petiole. Unlike *NAC006*, three genes, *NAC020*, *012*, and *071*, were only expressed in the cotyledon. The leaf was the most important organ, and its phenotypes were easily observed after stress treatment. We selected the 10 most highly expressed genes—*NAC003*, *016*, *025*, *034*, *043*, *045*, *060*, *065*, *070*, and *078*—for further research.

### 2.6. qRT-PCR Analysis of the NAC Genes

**Figure 5:**
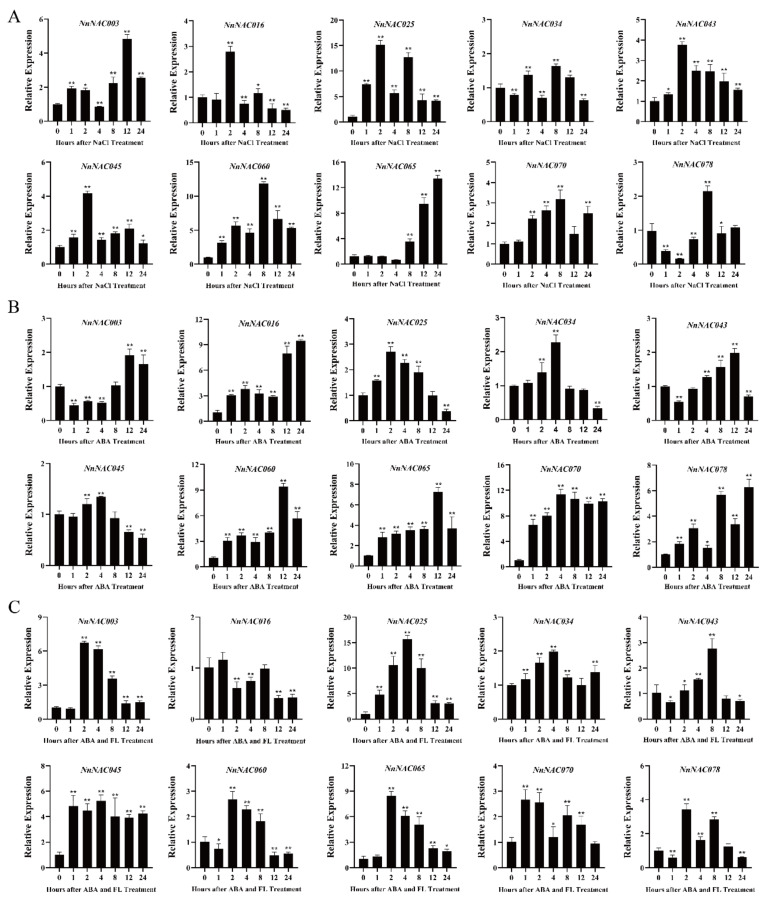
Relative expression levels of lotus NAC genes. qRT-PCR analyses of plants treated with NaCl (**A**), ABA (**B**), and ABA + FL (**C**). Independent *t*-tests demonstrated that there was significant difference (* *p* <0.05, ** *p* < 0.01).

To investigate the functions of the *NAC* genes, the above selected genes were analyzed by qRT-PCR under NaCl, ABA, and ABA + FL treatment (Figure 5). Under NaCl treatment, the transcription levels of nine *NAC* genes increased gradually, with *NnNAC078* being the sole exception. Under ABA treatment, the 10 *NAC* genes were all induced, and the transcription level of *NnNAC003* fluctuated. Fluoridone, the inhibitor of ABA, also induced gene transcription. In most cases, FL affects the action of ABA. In addition, the interaction of ABA and FL had positive effects on the expression levels of NAC genes including *NnNAC003*, *NnNAC025*, *NnNAC045*, and *NnNAC065*. The expression levels of *NnNAC16*, *NnNAC25*, and *NnNAC70* were significantly increased under ABA and NaCl treatment, while their expression levels were significantly reduced under ABA and FL treatment; they were selected for further research.

### 2.7. Subcellular Localization of NAC Proteins

**Figure 6:**
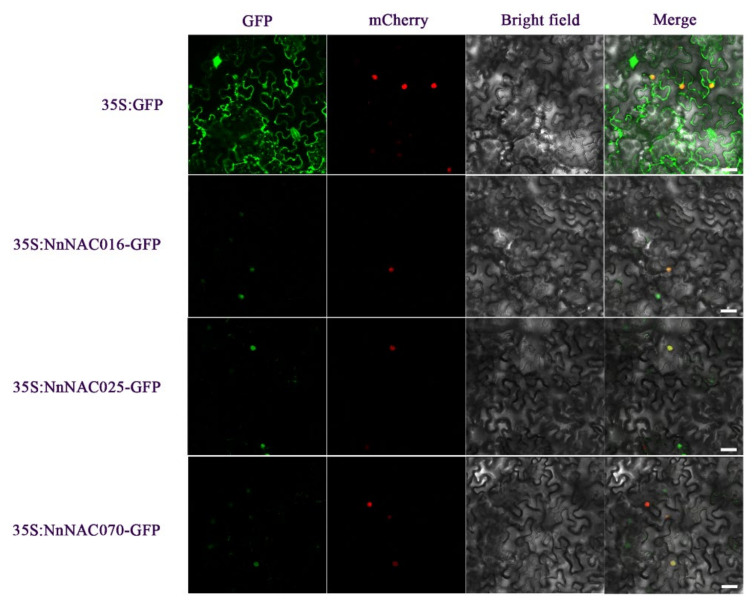
Subcellular localization of the soybean NAC proteins. The first row of pictures above is a control, and those below it are the NnNAC016, NnNAC025, and NnNAC070 proteins. Scale bars = 25 μm.

Subcellular localization could indicate the role and position of NAC-encoded proteins. In order to understand the molecular characteristics of hormone- and stress-responsive lotus root NACs, we selected three lotus root NAC genes for subcellular localization. Green fluorescence of the NnNAC16, NnNAC25, and NnNAC70 proteins all appeared in the nucleus, while green fluorescence of the control protein appeared on the cell membrane, in the cytoplasm, and in the nucleus, which indicated that the NnNAC16, NnNAC25, and NnNAC70 proteins function in the nucleus (Figure 6).

### 2.8. The Transcriptional Activation Capabilities Analysis of NAC Proteins

**Figure 7:**
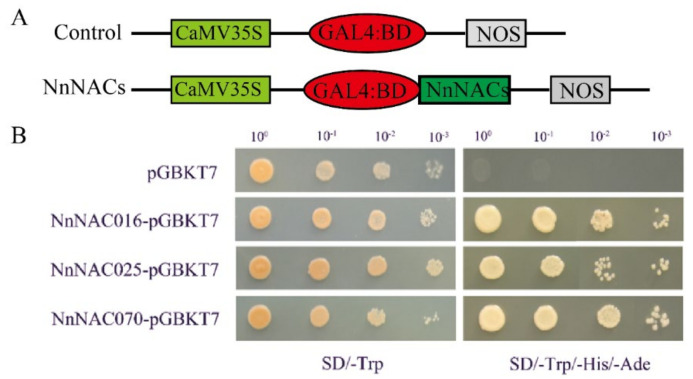
Transcription activation analysis of the three selected ANC proteins. (**A**) The diagram shows the structure of the fusion plasmid. (**B**) Transcription activation analysis of the nine selected NAC proteins. pGBKT7 was used as a negative control.

Transcription factors generally have self-activation ability. The full-length cDNAs of *NnNAC16*, *NnNAC25*, and *NnNAC70* were cloned into a PGBKT7 vector, and the self-activation ability of NnNAC16, NnNAC25, and NnNAC70 was verified by yeast experimentation. The results showed that NnNAC16, NnNAC25, NnNAC70, and the negative control pGBKT7 could all grow colonies on an SD/-Trp medium, while only the negative control pGBKT7 failed to grow colonies on an SD/-Trp/-Ade/His medium. This indicated that NnNAC16, NnNAC25, and NnNAC70 all had self-activation ability (Figure 7).

To further investigate the functions of the three *NnNAC* genes, their homologs in *Arabidopsis* were identified. Then, their co-expression networks were drawn using the website STRING. The results indicated that the functions of NAC genes were diverse and closely connected with NaCl, drought, and the plant hormone ABA (Figure S2). Co-expression networks allowed us to better study NAC genes in lotus root.

## 3. Discussion

With the completion of the genome sequencing of lotus root, several gene families were identified [44,47,53]. NAC proteins have diverse functions and play vital roles in plant growth and development, including responses to biotic and abiotic stresses [14,17,54,55,56,57,58,59,60]. However, functional research of lotus *NAC* genes has rarely been reported. Eighty-two NAC genes were obtained using the website PlantTFDB, and we studied their potential functions in lotus seeds and response to complete submergence [61]. Compared to NAC genes of other plants, the number NAC genes in lotus root still needs to be found. Here, we identified 97 NAC gene members by searching the whole lotus genome based on the raw HMM models for the conserved NAM domain (PF02365) and NAC domain (PF01849), which could look for as many genes as possible. Then, the 97 members were divided into 18 subgroups based on the classification method used in other plants [62,63]. The promotor, as an important structure, plays vital roles in plant growth and development. All NAC genes had the “light responsiveness” element, which is the fundamental element for plant growth and development. ABA is an important phytohormone regulating plant growth, development, and stress responses [64]. The “abscisic acid responsiveness” element in the promotor of *NAC* genes indicated that *NAC* genes may be regulated by the ABA-related transcription factors. Meanwhile, the qRT-PCR analysis also showed that *NAC* genes were induced under ABA treatment (Figure 4). In addition, the co-expression network of *Arabidopsis* homologs of three *NnNAC* genes also indicated the functions of *NAC* genes and ABA signaling are closely connected (Figure 7). For example, PwNAC11 activated *ERD1* by interaction with ABA-related proteins, ABF3 and DREB2A, to enhance drought tolerance in transgenic *Arabidopsis* [65]. *OsNAC45* was involved in ABA response and salt tolerance in rice [38]. In addition, “gibberellin responsiveness”, “MeJA responsiveness”, “salicylic acid responsiveness”, and “auxin responsiveness” were also widely presented in *NAC* gene promotors, which indicates NAC genes are multifunctional, interacting with many plant hormones’ signaling. These could provide us with a new avenue by which to study the function of *NAC* genes. Previous research showed that MeJA enhanced ethylene synthesis by inducing *NAC* genes in kiwifruit [66]. In addition, NAC genes could function in response to biotic stresses based on the “defense and stress responsiveness” element in their promotors. In total, the functions of NAC genes still need to be further studied.

The lotus is an important ornamental, edible, and medicinal plant originating from India and China. It is famous for its products such as fresh rhizomes, lotus root starch, drinks, teas, and lotus seeds [67]. However, the plant’s yield and product quality are affected by the salinity of soil [68]. Therefore, to excavate the key resistance genes is an important work for breeding superior lotus varieties. Furthermore, our results showed that NAC genes could be candidate genes to improve lotus resistance to slat stress. For instance, *NnNAC003*, *NnNAC016*, *NnNAC043*, *NnNAC045*, *NnNAC060*, and *NnNAC070* were significantly induced under NaCl treatment (Figure 4). Further results indicated that their functions in response to salt stress were dependent on ABA signaling (Figure 3 and Figure 4). In order to understand where NAC proteins function, we conducted a subcellular localization assay which demonstrated that NAC proteins function in the nucleus, as do most transcription factors (Figure 5). Like most transcription factors, the three NAC proteins, NnNAC16, NnNAC25, and NnNAC70, have transcriptional activation, which could bind them to the related *cis*-elements to regulate the expression levels of target genes. In addition, NAC proteins may form complexes with other proteins to play a role together (Figure 7). 

In total, the 97 *NAC* genes may be involved in responses to biotic and abiotic stresses by depending on ABA or other plant hormone signaling and would be the key candidate genes for further functional research by modern genetic and biological techniques.

## 4. Materials and Methods

### 4.1. The Identification of NAC Transcription Factor Members

The *Nelumbo nucifera* Geartn. genome (China Antique v2.0) was obtained from the Nelumbo Genome Database [48]. The raw HMM models for the conserved NAM domain (PF02365) and NAC domain (PF01849) were downloaded from the Pfam database (https://pfam.xfam.org/ (accessed on 27 May 2022)). the raw HMM models were then used to search the *Nelumbo nucifera* Geartn. genome with the software TBtools [52] to obtain the candidate NAC transcription factor members. Then, certain NAC transcription factor members were determined by their sequence search in the Pfam database with the threshold of an e-value <e−5. Next, to exclude repeated sequences, all NAC sequences were aligned using DANMAN Version 7 and checked manually. 

### 4.2. Basic Information and Phylogenetic Analyses

The NAC genes were visualized on the chromosomes using the software TBtools [52]. Their CDS length, molecular weight, and isoelectric point were predicted by the ExPASy tool (https://www.expasy.org/ (accessed on 5 June 2022)). The amino acid sequences of *Arabidopsis* and *Nelumbo nucifera* Geartn. were download from TAIR (https://www.arabidopsis.org/ (accessed on 5 June 2022)) and the Nelumbo Genome Database [48], respectively. Based on their amino acid sequences, the phylogenetic tree was constructed in MEGA7.0 by the neighbor-joining (NJ) algorithm; 1,000 replicates were used to evaluate the significance of nodes [69]. The annotation on the phylogenetic tree was created in the Adobe Illustrator SC5 software.

### 4.3. Synteny and Gene Structure Analyses

The applet MCScanX (default parameters) in TBtools was used to examine duplicate genes. Then, the homology of the NAC gene between *Nelumbo nucifera* Geartn. and Arabidopsis was visualized using the applet Dual Synteny Plotter in TBtools [52]. The conserved motifs of NAC proteins were identified using the online tool MEME Suite. The cis-element of the NAC gene promotor was analyzed via the website PlantCARE [70]. The motifs and cis-element were exhibited in one figure by TBtools.

### 4.4. Microarray Analysis of NAC Gene Expression Patterns

The tissue expression data of NAC genes was downloaded from the Nelumbo Genome Database. The analysis was carried out, which included the 97 NAC genes in the different tissues and development stages, and then it was visualized by TBtools.

### 4.5. Subcellular Localization Analysis

The selected proteins were fused with the expression vector pCAMBIA1300, containing a GFP tag for subcellular localization analysis, as described previously [71]. A diluted agrobacterium solution (NAC-pCAMBIA1300 or pCAMBIA1300) was injected into the leaves of tobacco. After 24 h of incubation, fluorescence images were captured by a confocal laser scanning microscope (TCS SP8; Leica). The primers used in this study can be found in Table S1.

### 4.6. Plant Materials and Treatments

Lotus seeds were germinated in soil in a greenhouse. Two-week-old seedlings were subjected to exogenous ABA and salinity treatments. For these treatments, seedlings were transferred to solutions containing 200 mM NaCl or 100 μM ABA. Seedlings were sampled at 0, 1, 2, 4, 8, 12, and 24 h after treatment. The samples were then dropped immediately into liquid nitrogen and stored at −80 °C.

### 4.7. Quantitative Real-Time PCR (qRT-PCR)

The samples were ground in liquid nitrogen, and the powder was used to extract the RNA. The cDNA synthesis was conducted following the manufacturer’s instructions using a PrimeScript™ 1st Strand cDNA Synthesis Kit (TaKaRa, Beijing China). Then, the expression patterns were analyzed with a BIO-RAD CFX MaestroTM system. In this assay, three biological and technical replicates were made. The histogram was made with the GraphPad Prism 8.0 software, and the standard error was calculated with the SPSS software. The primers used in this study can be found in Table S1.

### 4.8. The Transcriptional Activation Assays

The transactivation ability of NAC TFs was tested in yeast cells. The fusion plasmids NnNACs:pGBKT7 and the control plasmid pGBKT7 were transformed into Y2H Gold yeast cells. Transformed yeast competent cells were spread on plates containing SD/-Trp or SD/-Trp/-His/-Ade.

## Figures and Tables

**Table 1:** The *NAC* genes and properties of the deduced proteins in lotus root.

Gene Name	Gene ID	Genomic (bp)	Transcript (bp)	CDS (bp)	Amino Acid Residues (aa)	p*I*	MW (KDa)
*NnAC001*	Nn1g00014	808	571	571	190	4.48	21.73
*NnNAC002*	Nn1g00424	1478	1219	1219	406	6.78	38.7
*NnNAC003*	Nn1g00471	3868	1768	1768	589	9.59	68.3
*NnNAC004*	Nn1g00574	2293	1147	1147	382	5.16	43.21
*NnNAC005*	Nn1g01090	3088	1138	1138	379	6.35	41.38
*NnNAC006*	Nn1g02798	4088	847	847	282	9.38	19
*NnNAC007*	Nn1g03472	825	628	628	209	9.44	24.45
*NnNAC008*	Nn1g03986	2380	1171	1171	390	9.29	43.13
*NnNAC009*	Nn1g03987	549	286	286	95	7.01	41.53
*NnNAC010*	Nn1g04183	2056	1279	1279	426	6.25	48.01
*NnNAC011*	Nn1g04185	1898	1150	1150	383	6.57	43.76
*NnNAC012*	Nn1g04206	1652	1069	1069	356	8.36	39.32
*NnNAC013*	Nn1g04246	1929	973	973	324	7.79	41.69
*NnNAC014*	Nn1g04798	38,082	772	772	257	7.66	38.71
*NnNAC015*	Nn1g05100	4004	430	430	143	4.76	30.56
*NnNAC016*	Nn1g05179	2747	1675	1675	559	4.89	63.62
*NnNAC017*	Nn1g06246	8935	1390	1390	463	4.93	51.29
*NnNAC018*	Nn1g06289	811	571	571	190	4.48	21.82
*NnNAC019*	Nn1g06403	2271	964	964	321	6.25	26.5
*NnNAC020*	Nn1g06441	2864	1033	1033	344	8.01	37.92
*NnNAC021*	Nn1g06666	2070	841	841	280	9.16	44.2
*NnNAC022*	Nn1g06671	1365	1063	1063	354	8.89	40.32
*NnNAC023*	Nn1g08315	1708	1279	1279	426	6.13	48.21
*NnNAC024*	Nn1g08339	1770	1282	1282	427	6.13	48.28
*NnNAC025*	Nn1g08874	907	907	907	302	8.39	34.63
*NnNAC026*	Nn2g12755	7485	949	949	316	8.77	35.3
*NnNAC027*	Nn2g13806	1016	649	649	216	4.93	26.4
*NnNAC028*	Nn2g13861	1328	820	820	273	4.64	62.23
*NnNAC029*	Nn2g14216	1499	622	622	207	9.34	23.41
*NnNAC030*	Nn2g14870	1290	532	532	177	9.15	21.13
*NnNAC031*	Nn2g14874	1991	1012	1012	337	6.07	38.87
*NnNAC032*	Nn2g15917	1522	1246	1246	415	9.46	23.88
*NnNAC033*	Nn2g15921	3819	1153	1153	384	8.48	43.41
*NnNAC034*	Nn3g16348	922	229	229	76	9.86	18.71
*NnNAC035*	Nn3g16672	348	349	349	116	8.91	13.64
*NnNAC036*	Nn3g16695	9610	1183	1183	394	5.6	43.89
*NnNAC037*	Nn3g17225	2541	1255	1255	418	6.66	46.29
*NnNAC038*	Nn3g17296	1617	1069	1069	256	8.99	33.49
*NnNAC039*	Nn3g17297	1123	847	847	282	6.26	32.37
*NnNAC040*	Nn3g17722	14,012	1480	1480	493	5.09	55.55
*NnNAC041*	Nn3g17854	1580	1009	1009	336	7.54	37.46q
*NnNAC042*	Nn3g18857	1031	1222	1222	407	6.89	46.14
*NnNAC043*	Nn3g19685	4659	850	850	283	5.58	31.98
*NnNAC044*	Nn3g21809	1945	349	349	116	9.49	13.55
*NnNAC045*	Nn4g22208	1379	775	775	258	9.38	29.22
*NnNAC046*	Nn4g22597	7186	1354	1354	451	5.5	50.44
*NnNAC047*	Nn4g23151	1916	1234	1234	411	8.25	45.91
*NnNAC048*	Nn4g23226	1304	1072	1072	357	7.05	40.27
*NnNAC049*	Nn4g23228	816	478	478	159	9.41	18.61
*NnNAC050*	Nn4g23242	1085	847	847	282	6.67	32.54
*NnNAC051*	Nn4g23388	701	514	514	171	6.66	20.03
*NnNAC052*	Nn4g23853	1406	904	904	301	7	34.07
*NnNAC053*	Nn4g24534	372	373	373	124	9.98	14.2
*NnNAC054*	Nn4g24728	4125	2053	2053	684	5.31	77.94
*NnNAC055*	Nn4g24729	2437	715	715	238	5.06	27.26
*NnNAC056*	Nn4g24873	2218	1054	1054	□	8.84	40.27
*NnNAC057*	Nn4g25922	1642	460	460	153	9.92	18.13
*NnNAC058*	Nn4g25937	1644	460	460	153	9.7	18.1
*NnNAC059*	Nn4g26681	2233	964	964	321	7.69	37.02
*NnNAC060*	Nn5g27114	2326	481	481	160	9.04	18.97
*NnNAC061*	Nn5g27174	4421	1081	1081	360	8.13	40.74
*NnNAC062*	Nn5g27709	1144	943	943	314	8.97	19.65
*NnNAC063*	Nn5g28365	394	490	490	163	5.84	34.06
*NnNAC064*	Nn5g28509	1373	592	592	197	7.7	21.99
*NnNAC065*	Nn5g28579	9445	1387	1387	462	5.03	46.52
*NnNAC066*	Nn5g28895	3086	1705	1705	568	9.53	61.43
*NnNAC067*	Nn5g29253	4770	1027	1027	342	5.37	38.58
*NnNAC068*	Nn5g29499	3786	397	397	132	5.81	45.6
*NnNAC069*	Nn5g30346	8202	658	658	219	9.53	22.18
*NnNAC070*	Nn5g30910	2903	895	895	298	8.55	29.31
*NnNAC071*	Nn5g31036	2104	1111	1111	370	5.74	41.69
*NnNAC072*	Nn6g31660	2301	1156	1156	385	6.36	42.44
*NnNAC073*	Nn6g31768	1749	772	772	257	8.59	35.64
*NnNAC074*	Nn6g32265	2062	1000	1000	333	5.77	38.39
*NnNAC075*	Nn6g33629	3148	271	271	90	9.44	29.17
*NnNAC076*	Nn6g34050	2482	1099	1099	366	6.81	42.52
*NnNAC077*	Nn6g34819	1205	760	760	253	6	28.76
*NnNAC078*	Nn6g34820	1464	793	793	264	6.8	30.26
*NnNAC079*	Nn6g35218	16,416	1771	1771	590	4.66	54.24
*NnNAC080*	Nn6g35359	3153	874	874	291	6.6	47.95
*NnNAC081*	Nn7g35779	1087	913	913	304	5.38	34.73
*NnNAC082*	Nn7g37118	1575	1015	1015	338	5.46	38.78

## Data Availability

Not applicable.

## Supplementary Materials

The supporting information can be downloaded at: https://www.mdpi.com/article/10.3390/ijms232012394/s1.
