# Supplementary figures and images for: Identification of the NAC Transcription Factors and Their Function in ABA and Salinity Response in Nelumbo nucifera
Source: Int J Mol Sci. 2022 Oct 16;23(20):12394. doi: 10.3390/ijms232012394 (PMC9604248; doi:10.3390/ijms232012394)

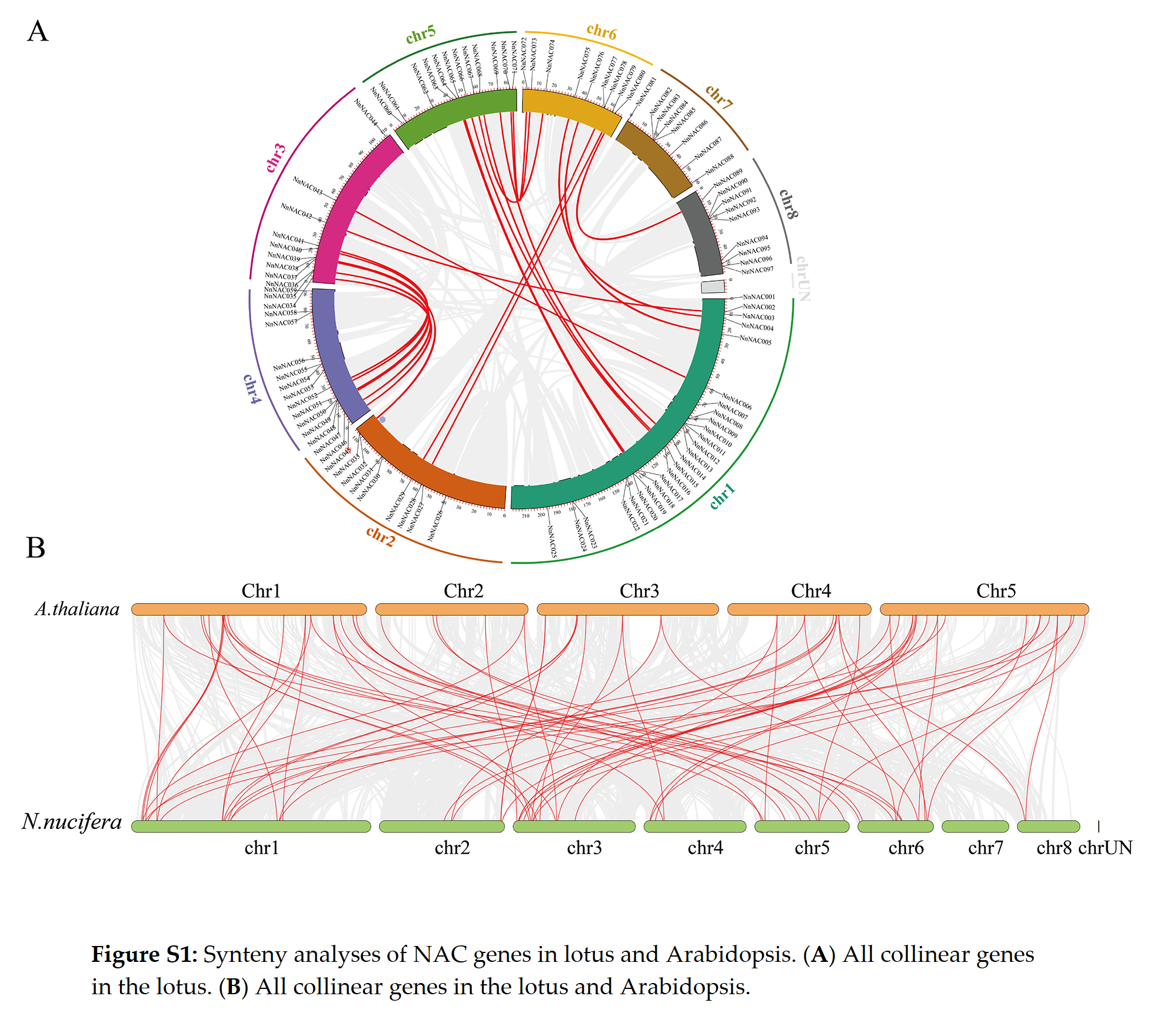

Supplement: Supplementary file 1 [file ijms-23-12394-s001.zip › Figure S1.tif]

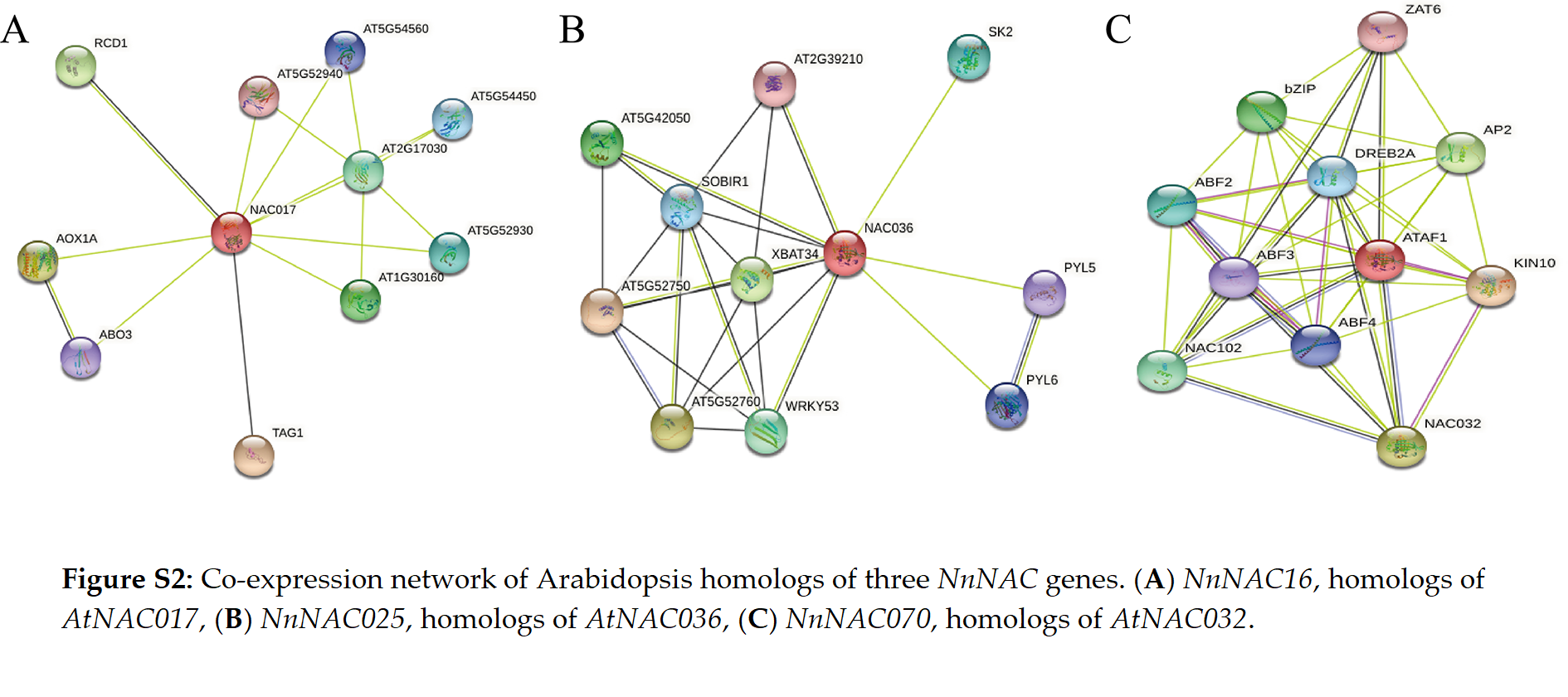

Supplement: Supplementary file 1 [file ijms-23-12394-s001.zip › Figure S2.tif]
